# Moral Distress in Community and Hospital Settings for the Care of Elderly People. A Grounded Theory Qualitative Study

**DOI:** 10.3390/healthcare9101307

**Published:** 2021-09-30

**Authors:** Giulia Villa, Federico Pennestrì, Debora Rosa, Noemi Giannetta, Roberta Sala, Roberto Mordacci, Duilio Fiorenzo Manara

**Affiliations:** 1 Center for Nursing Research and Innovation, Vita-Salute San Raffaele University, Via Olgettina 58, 20132 Milan, Italy; villa.giulia@hsr.it (G.V.); giannetta.noemi@unisr.it (N.G.); manara.duilio@unisr.it (D.F.M.); 2 Faculty of Philosophy, Vita-Salute San Raffaele University, Via Olgettina 58, 20132 Milan, Italy; pennestri.federico@unisr.it (F.P.); sala.roberta@unisr.it (R.S.); mordacci.roberto@unisr.it (R.M.); 3 Department of Cardiovascular, Neural and Metabolic Sciences, Istituto Auxologico Italiano-IRCCS, Piazzale Brescia 20, 20149 Milan, Italy

**Keywords:** bioethics, caregiver, decision making, elderly, grounded theory, hospital, moral distress, nursing home

## Abstract

*Background:* Moral distress has frequently been investigated in single healthcare settings and concerning a single type of professional. This study aimed to describe the experience of moral distress in all the types of professionals providing daily care to elderly patients and residents. *Methods:* The Grounded Theory approach, developed by Corbin and Strauss, was used. This study included participants from hospital and nursing homes of northern Italy. Purposive and theoretical sampling was used. Between December 2020 and April 2021, semi-structured interviews were conducted. *Results:* Thirteen participants were included in the study. Four categories were derived from the data: talking and listening, care provider wellbeing, decision making, protective factors, and potential solutions. The core category identified was “sharing daily”. Interviewees confirm how hard it may be to communicate to the elderly, but at the same time, how adequate communication with the leader is a protective factor of moral distress. They also confirm how communication is key to managing or downsizing misunderstandings at all levels. Findings highlight the scarcity of operators as a fundamental trigger of moral distress. *Conclusions:* Many determinants of this phenomenon lie behind the direct control of professionals, but education can help them learn how to prevent, manage, or downsize the consequences.

## 1. Introduction

The concept of moral distress (MD) was introduced in 1984 by Jameton, to describe the psychological distress of being in a situation in which a healthcare professional is constrained from acting on what he/she knows to be right [1]. Conflicting emotions are common in those professions based on the permanent contact with people, as many types of environmental constraints (from prudential reasons to physical barriers) can prevent the operators from acting and reacting as they would. Many studies investigate the phenomenon in different professions: social workers, who may suffer the pressure of political institutions [2], may need to “push the rules”, or go “in through the back door” to provide their patients the assistance and/or resources they need [3]; lawyers and attorneys, who frequently need to face conflicting ethical decisions [4] and may choose to offer their service depending on the influence and revenue of clients [5]; school teachers, who can find themselves stuck between following their own values and meeting hierarchical orders of a prudential nature [6,7,8], with specific tools being dedicated to measure their levels of distress [9]; and call center operators, who may desire to react differently to the people they talk to in comparison to what is required by their employers [10].

Sometimes, the inability to act on the patient’s interest can lead to undertaking difficult ethical decisions and experience frustration [11], which may trigger—in turn—psychophysical problems such as pain and lack of balance, resulting in burnout, blame, reduced safety and quality of care both in the long and the short term [12,13,14,15,16]. Moral distress and burnout received particular focus in relation to health care professionals, as they spend most of their time providing care to people who are vulnerable by definition and place further pressure on their decisions [17,18]. Although MD received particular attention among nurses [16,19], physicians [14,20], physical therapists [21], and further healthcare assistants are also exposed to the same risks [22]. Feelings associated with the experience of MD include anger, guilt [23], and helplessness [24], which seem to occur more frequently during the early years of employment, where a discontinuity between the ideals of the profession and the harshness of daily work is often perceived [20,25,26,27,28].

These challenges become more demanding when the patient is elderly, as poor psychophysical autonomy and different degrees of cognitive dysfunction can undermine his/her ability to understand [29,30], take, and maintain decisions that affect their health [31,32,33]. Population aging is a long-term trend that began several decades ago in Europe, and the number of older people is expected to double to 1.5 billion in 2050 [34]. Among the members of the European Union, in the year 2019, 22.8% of the population aged 65 and over were Italian. Therefore, Italy is particularly sensitive to this trend: nearly 2.1% of the elderly people are hosted in social care facilities and about 210,000 are not self-sufficient [34].

MD has frequently been investigated in single healthcare settings and concerning a single type of professional [35,36]. This study aims to analyze the experience of MD in all types of professionals providing daily care for elderly patients and residents, including both acute and long-term care facilities. The setting is a region of northern Italy, the Lombardy, in which aging is particularly pronounced, and greater investments in community care are being implemented [37]. At the same time, this analysis is expected to generate new knowledge about how the participants handle a morally distressing event and how their actions and choices are limited or enhanced by environmental triggers (time, space, relations, or other factors).

## 2. Materials and Methods

The study design was a Grounded Theory approach developed by Corbin and Strauss [38,39]. The analysis of the qualitative data was conducted using the Grounded Theory method in the version of Strauss and Corbin [38].

### 2.1. Sampling 

Participants had to meet the following inclusion criteria: working experience providing care for the elderly for more than six months; willingness to participate in the study; knowledge and understanding of the Italian language. Exclusion criteria were non-professional caregivers, students, or trainees. Participants were recruited by convenience sampling, and subsequently, after additional knowledge on moral distress was learned, by theoretical sampling [40]. The study population confirmed the intention to partake in the interviews by leaving their name and contact (telephone and/or email) between November 2020 and February 2021. Subsequently, these individuals were contacted by the same researcher who conducted the interviews (DR) and recruitment, until data saturation was reached [41,42]. Thirteen participants met the inclusion criteria and were enrolled in the study. Among them, six came from a hospital setting in northern Italy and seven from two nursing home organizations in two provinces in northern Italy. The socio-demographic characteristics of the participants are described in Table 1.

### 2.2. Structure of the Interviews

In-depth, semi-structured interviews with an average duration of 32 min (range: 21–45) were conducted between December 2020 and April 2021. The interviews contained a list of topics and issues for discussion. The interview consisted of open-ended questions aimed at understanding how participants deal with a morally distressing event and explore how their actions and choices are limited or enhanced by environmental triggers. At the beginning of each interview, the researcher required all participants to describe only experiences prior to the COVID-19 pandemic.

The interviews were conducted by a female researcher (D.R.), Ph.D. in Nursing and Public Health, with experience in qualitative research and working as a nursing researcher. The research team also consisted of one Associate Professor in Nursing, two researchers with a Ph.D. in Nursing and Public Health, one researcher with a Ph.D. in Philosophy and Mind Sciences, and two Full Professors in Ethics and Philosophy, who contributed to the supervision of the paper and the interpretation of results.

During the interviews, a researcher participated as a third observer (G.V., N.G.) to collect field notes [41,43]. Each participant was interviewed individually after obtaining informed consent and using videoconferencing platforms (Skype, Teams, Zoom) [44]. Interviews were video-recorded, with verbal consent from participants, and subsequently transcribed verbatim. Accuracy and rigor were ensured by the cross-checking of two researchers: the former taking care of the transcription of the collected data, the latter listening to the audio recording, while reading the transcription, in order to ensure that it reflected the words of the interviewee. Any information that could have made the interviewee recognizable was edited to protect his or her identity. For this purpose, pseudonyms were used instead of names of people, cities, streets, and organizations [41,43].

## 3. Results

**Figure 1:**
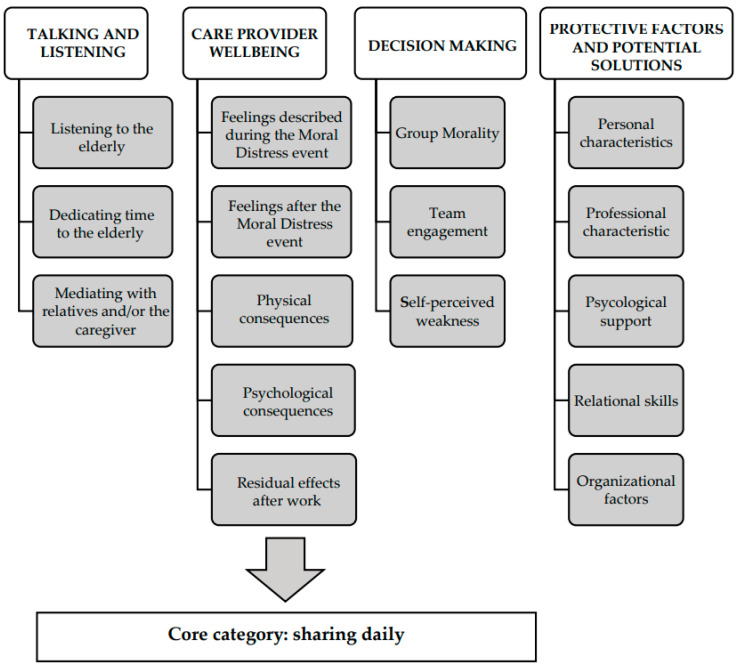
Category and sub-category.

The data are described through categories and sub-categories, building on the theoretical framework proposed by Corbin and Strauss [40,44]. The categories include: (1) talking and listening, (2) care provider well-being, (3) decision-making, and (4) protective factors and potential solutions. The categories were aggregated into the core category of “sharing daily”. The sub-categories are detailed in Figure 1.

### 3.1. Talking and Listening

Three sub-categories were extracted from the category “talking and listening”: listening to the elderly; dedicating time to the elderly; mediating with relatives or the caregiver. 

#### 3.1.1. Listening to the Elderly

Talking with the operators is a fundamental need presented by the elderly.

“From the human point of view [older people] have relationship needs because often [they are] lonely people and so they often want to chat and to exchange ideas, opinions; to tell each other. And, they need attention.”

[ID 6]

“Someone to explain to them how to cope with life with their illness.”

[ID 3]

“Sometimes, for example, the patient would ask me to simply spend some time together, for some company.”

[ID 2]

#### 3.1.2. Dedicating Time to the Elderly

The impossibility to dedicate time to the elderly, more than fulfilling technical obligations alone, can be frustrating for professional caregivers.

“An elderly man was, unfortunately, going to die, and I could not devote time to him. I was not close to him in such a delicate moment, when he was alone, no family and no caregiver.”

[ID 1]

“My crisis is determined by the fact that the team in which I work, has many difficulties in treating people with these problems [aggressiveness]. However, I felt that in this case there was the possibility to do something more. In my opinion, if we had to go beyond the protocols that our company requires, I think we could have had better success. So, I feel a bit guilty that I was not able to make others understand that there are alternative ways of dealing with such serious problems.”

[ID 11]

#### 3.1.3. Mediating with Relatives and/or the Caregiver

Difficult communication with relatives or caregivers can trigger frustration and MD, especially when the good of the elderly patient or resident is misaligned between the same relatives and the single care providers: at the clinical level, about the procedures to be performed; at the organizational level, about the respect of the ward timetable; and the emotional levels, concerning the positive, constructive, or catastrophizing reactions presented by the relatives.

“One of the situations in which we often find ourselves is having a relative who pressurizes us and says for example: ‘let’s do everything we can’, and then, the elderly person who tells you, for example, I don’t want to dialyze anymore, so you find yourself having the elderly person who is there, maybe rather weakened [by illness], that you have to tie him up, sedate him to do the dialysis session, because the patient anyway is dangerous if he tears out the needles, in the end, he bleeds to death.”

[ID 3]

“It can happen that the patient’s daughter arrives, and she pretends that daddy comes down with her to have coffee, while there was a very precise program with a physiotherapist, then the patient had to have at 10 o’clock antibiotic and sometimes everything is a bit difficult for her to understand.”

[ID 4]

“I also do the video calls. I have this appointment with a lady and her daughter on dementia, so two pathologies together. Over the months she has clearly worsened and therefore her daughter, unfortunately, on the phone, is often in difficulty, she cries, etc. and any talk, in short, is useless.”

[ID 10]

### 3.2. Care Provider Well-Being

The impact of MD on health and social care providers was classified into four sub-categories: feelings described *during* the MD event; feelings described *after* the moral distress event; physical consequences; psychological consequences; residual effects after work.

#### 3.2.1. Feelings Described during the MD Event

Helplessness and fear of consequences were associated with the occurrence of the MD event.

“I felt the weight on my shoulders of what was happening, and I was alone. I was alone and he was alone. I mean he was alone in the sense that he didn’t have anybody, and I was alone because I had so many other patients.”

[ID 1]

“There is fear, there is also fear it is latent it is not perceived no...”

[ID 5]

#### 3.2.2. Feelings after the MD Event 

Controversial feelings were described after the decision that generated MD. However, satisfaction was felt once the right action was performed.

“I felt I was doing the right thing [leaving some nursing activities behind, to be with the patient who was dying alone].”

[ID 2]

“I felt, I had confirmation of the goodwill and correctness of the method.”

[ID 5]

On the other hand, failure, and overthinking:

“It happened to me [within the group, the team] to be the only one who thought that it was necessary to think about it more, maybe postpone an exam [for the elderly person] or not do it.”

[ID 6]

“Of failure. I felt failed unable to even make others listen to me, so a general inability. Almost, almost I questioned my professional skills because if I was not able to convince others of the usefulness of what they were proposing I told myself maybe I was not so convincing.”

[ID 11]

#### 3.2.3. Physical Consequences

Few respondents stated that they had experienced physical problems, although sometimes irreversible.

“It changed my life drastically because it made me hypertensive at the age of 40.”

[ID 5]

“So, the conclusion I’m aware of hurting myself and neglecting myself because anyway the translation of the fatigue of it all was obviously to make personal life choices that undoubtedly neglected my health.”

[ID 5]

“I had headaches sometimes, but not so much because of a relationship with the host, but perhaps because of the demands of the organization that became excessive, let’s say, and therefore making people understand that they become excessive sometimes gives them a headache.”

[ID 10]

#### 3.2.4. Psychological Consequences

A couple of respondents reported psychological consequences attributable to distraction and isolation, both at work and at home.

“When I came home, I was very distracted. I had to talk to my boyfriend and so on, but I was completely somewhere else. I wasn’t connecting, I was just distracted.”

[ID 1]

“It causes me a sense of depression, crying, closure towards relationships with others.”

[ID 11]

#### 3.2.5. Residual Effects after Work

The respondents reported different attitudes toward speaking about MD events at home or not, telling of being able to hold back their worries deriving from work, depending on both individuals’ attitudes and the people they are close to (i.e., colleagues or not; people experiencing similar situations in their own life or not).

“At home, I bring back practically everything. Every time I arrive home and I am a blackboard. I always have to say everything, in fact, that’s what I can’t, that is why I cannot many times detach myself. I sometimes cannot switch off when I am at work, professionally.”

[ID 7]

“I think that a person who is not confronted with these situations cannot in my opinion understand much.”

[ID 1]

“With friends sometimes, we respect the sense of some things maybe without entering into the specific... because maybe, I find that they have to manage relatives.”

[ID 3]

### 3.3. Decision Making

Factors affecting the decision-making process of professionals taking care of the elderly were divided into three sub-categories: group morality; team engagement; self-perceived weaknesses.

#### 3.3.1. Group Morality

One interviewee referred to group morality as acting on a shared decision in front of the patient and/or their family. Although each member of the team, individually, may have a different course of action in mind, relying on group morality was considered a positive strategy to reduce misunderstandings among colleagues, patients, and their relatives.

“Having a group moral, because one can have one’s own opinion. It’s one of the things that is most disorienting for the patient and the relatives, to see conflicting opinions, so I am one who believes anyway that in the end, you have to follow the will of the leader even if you do not agree, it is something that I think is right, this concept, so I do it.”

[ID 3]

“If the team has decided that there was something to be done, I accept the result also because we bring something home.”

[ID 6]

“Based on the assumption that my way of dealing with these patients who have issues, that also goes to affect the work of others.”

[ID 12]

#### 3.3.2. Team Engagement

Several respondents shared their feelings and choices with colleagues and the team.

“I have only spoken with colleagues because they have experienced the situation with me...”

[ID 1]

“[The right choice] has always been a collective choice, it has always been a choice so in communion with colleagues there is no fundamental choice of direction that has not been the product of consensus.”

[ID 5]

Other professionals and teams, however, are used to avoid any type of engagement.

“Nobody ever asked me or let me express myself. [No one] felt it was necessary for me to express how I felt about that problem, they just [told me] what I should do or what I should not do.”

[ID 11]

#### 3.3.3. Self-Perceived Weakness

Missing decision-making skills and being too emotionally involved were reported as self-perceived weaknesses when facing MD events.

“[I missed specific] Skills”

[ID 1]

“On the one hand, I felt maybe too much involvement in the situation i.e., that detachment that we should have at that time was maybe a bit lost.”

[ID 2]

“Probably related a little bit to the emotional aspect.”

[ID 10]

### 3.4. Protective Factors and Potential Solutions

Certain protective factors and potential solutions were identified by the participants to prevent or manage MD: personal characteristics; professional characteristics; support from the leaders; psychological support; relational skills; organizational factors.

#### 3.4.1. Personal Characteristics

Maintaining Soothing Behavior in Case of Distressing Events Was Reported Twice as a Protected Personal Characteristic, which Seems to Transmit Emphatical Benefits to the Other People Involved

“A strong point [during the MD event] is tranquility. If you are calm and you face problems in a serene way the other party also faces it serenely with the person.”

[ID 4]

“[My strength is] making others feel good so make them maybe smile and help them if they need it.”

[ID 7]

Faith was also mentioned twice, to make sense of a demanding profession and to endure the pressure of difficult decisions.

“I lived great ethical moments in my extra-professional life, as I grew up in an environment strongly oriented to attributing an ethical sense to what happens; not only in a religious environment but also in social terms: I participated in many non-religious volunteering activities in which the question was: what is the sense of what we are doing?”

[ID 5]

“When I have to make these decisions [whether or not to dialyze a patient who is now terminal] I think and rethink about it, I am religious I pray about it.”

[ID 3]

Finally, having a life rich in social relations was considered a protective factor, which also made psychological support useless.

“I never felt the need for psychological support. I do not say it with arrogance; but because my life is rich in social relationships.”

[ID 5]

#### 3.4.2. Professional Characteristics

Experience is a professional characteristic associated with the ability to manage demanding situations.

“Definitely the experience [is a protecting factor]. If it had happened to me the day after I started work, I would have panicked.”

[ID 12]

“[During the moral distress event] Neglect a little bit what is the real nursing work from the technical point of view.”

[ID 2]

#### 3.4.3. Support from the Leaders

Trust from managers, leaders, and the local administration was considered fundamental to support the harshness and dilemmas of daily work.

“A bit of a breath of benefit, of that light benefit of trust that sort of unconditional trust that the institution as authoritative wants: scientific director, my mentor, my professor, my medical director conferred in me.”

[ID 5]

“Good organization and good presence from the top.”

[ID 7]

#### 3.4.4. Psychological Support

Among the many respondents who recognized the utility of psychological support for care providers, one complained that this type of support is still considered taboo, as the need for psychological support would be the expression of an individual inadequacy.

“The nursing profession that has to have psychological support, because there are some jobs that ask for it, of course, and this is one thing that is a taboo in Italy. It is supposed if you say psychologist, it sounds as if you are crazy, but it is not like that.”

[ID 4]

#### 3.4.5. Relational Skills

Peculiar characteristics and needs of elderly patients and residents (receiving attention, being hosted for a lifetime, absorbing the transition from home) make it necessary to integrate technical cure (administering drugs, physical therapy, monitoring parameters, supporting the activities of daily living) with personal care (listening to the patient, asking him/her how he/she is, whether he/she slept well, spending a word of comfort); a fundamental skill to evolve from the former to the latter is to understand how the elderly explicitly or implicitly express their needs, or in other words, are responsive.

“I’m a big believer in personalized care.”

[ID 3]

“The key [to reduce MD] is the ability to include.”

[ID 5]

“[To reduce MD] you have to learn the language of older children.”

[ID 8]

#### 3.4.6. Organizational Factors

Hiring more operators and keeping each responsibility clear are organizational arrangements that may improve cooperation.

“Increasing the number of operators can be one thing that can influence a lot on this thing [preventing moral distress events].”

[ID 1]

“I am grateful to a structure that has always given me clarity of purpose.”

[ID 5]

“But in my opinion, it is the first thing to give everyone the gradual responsibilities they deserve.”

[ID 6]

### 3.5. Core Category

The core category identified was “sharing daily”:

“[What can reduce moral distress] is communication and sharing daily.”

[ID 5]

## 4. Discussion

This study aimed to analyze the experience of MD in all the types of professionals providing care to elderly patients and residents, including both acute and long-term care facilities, in a region and country in which aging is particularly pronounced [45]. At the same time, this analysis generated new knowledge about how the participants handle a morally distressing event and how their actions and choices are limited or enhanced by environmental triggers (time, space, relations, or other factors). The analysis of the qualitative data was carried out using the Grounded Theory method in the version of Strauss and Corbin [38]. In the present study, reliability was established by the detailed and descriptive analysis of the data and direct references to the individual’s professional experiences. The consistency of the analysis was maintained through meetings of the research team to discuss preliminary findings. The thematic analysis and coding process took place through consensus. To increase the transferability of the results, a description of the context, selection, and demographics of the participants, data collection, and analysis process were presented [41,43].

### 4.1. Scarcity of Operators vs. Responsiveness

Certain results confirm frequent experiences reported in the literature, while others add further knowledge or research cues. We may consider the scarcity of operators as a fundamental trigger of MD, as it may undermine the safety of patients and professionals (who in turn bear the additional pressure of this condition) and withdraws time from interprofessional and patient–caregiver communication [8]. “The breakdown of communication” is one of the most important determinants of errors and complications in the healthcare settings [45], with the following complications and legal complaints.

When taking care of the elderly, complex patients who have peculiar needs, there are ways to express their needs, and we appreciate those professionals who can meet them before they are expressed [46]. Responsiveness is a key skill—when facilitated by the workplace environment—to prevent further complications, improve the perceived quality of care, and distinguish real needs from whims. The peculiar needs reported in the sub-category “relational skills” (receiving attention, being hosted for a lifetime, absorbing the transition from home) clearly confirm what is known from the literature [46].

### 4.2. Hard Skills and Soft Skills

Education plays a fundamental role in highlighting, enhancing and training relational and reflective skills, introducing students to the peculiar challenges of their daily work. Therefore, not only is the education hard, technical skills remain a priority for safety and quality of care. In addition, the education of the so-called “soft skills” helps the operators to take the best advantage of technical skills for that patient they have in front, at that moment, in that place; communication is one of these skills [47,48]. Precisely when the operator recognizes the importance of personalized care, the impossibility to dedicate time to listen to the patient’s individual needs can undermine job satisfaction and quality of care. To consider each operator as the equivalent of another is similar to considering any patient as the equivalent of another. Being equivalent is something that organizations must carefully consider when they arrange shifts relying entirely on informative technologies and algorithms [49,50] as if the ward or the nursing home were an assembly line. It is not surprising, then, if the caring relationship between the operators and the elderly is described as “living in the same world without meeting” [51]. Furthermore, a life rich in social relations, both at work and outside, can be considered an equally effective antidote to psychological support.

### 4.3. Inclusion vs. Isolation

Inclusion, in this sense, can play a role in reducing the exposition to MD, according to one interviewee. The importance of positive social relationships becomes clearer when they are lacking, especially in particularly competitive workplace environments that favor the onset of self-blame among the operators, as if they were individually responsible for the poor quality of care [14]. Many determinants of MD lie behind the direct control of professionals, but education can help them learn how to prevent, manage or downsize the consequences, according to the authors, for instance, explaining the system-level determinants of MD and poor quality of care, such as limited resources, the “negative halo” surrounding eldercare [52], and higher expectations from patients and society. As such, these determinants are shared by all the professionals working daily with the elderly, to whom we may also add managers, regardless of any bias on individual fitness. That is why managers can be involved in these activities [53], provided there is a “consequence-free” environment [54].

### 4.4. External Pressure vs. Individual Balance

Conflicting demands from patients and family members [9] and patient opposition to care and assistance [55,56] have also resulted in MD. Instead, the empathic benefits potentially facilitated by tranquility and joy (in addressing their concerns) underline strategies that are almost neglected by the literature [57], to the best of the knowledge of the authors. Faith and spirituality can play a fundamental role in this purpose. Being able to make sense of the harshness of work may therefore be considered a protective factor to keep vocation alive and feel gratified, including in lack of (explicit) gratitude [58,59,60]. Personal balance and a solid philosophy of life are considered protective factors among nurses and medical doctors [61,62,63], including at the stage of education [64]. One interviewee reported how passion helped him to make sense of job challenges daily, to the extent of accepting and being aware of the physical consequences. This experience confirms both the correlation between MD and cardiovascular conditions of risk [65,66] and the adaptive, protective value of job satisfaction [67].

### 4.5. Sharing Daily vs. Accumulating Frustration

Many studies and reports confirm the importance of adequate communication across most of these situations. Communication is a central element to establish a relationship with the older person, with his/her relatives and/or caregiver, and with the team members. Confrontation is important both among peers and with leaders in the organization. Leaders who are not aware of what is going on can threaten the cohesion of the group, for instance, missing clear responsibilities and goals [53]. 

Our interviewees confirm how adequate communication with the leader is a protective factor, especially when combined with trust. The interviewees confirm how hard it may be to communicate to the elderly—talk to them, listen to them, mediate needs, priorities [46,68]. However, they also confirm how communication is key to manage or downsize misunderstanding at all levels (among colleagues, with leaders, with relatives and informal caregivers, with the same elderly patients and residents). This is confirmed by many contributions in literature. Primary care physicians receive fewer claims when their patients feel listened to [69]; nurses can accept more easily aggressive behaviors from patients when they are non-intentional [56], suspending the dichotomy between good and wrong, which is a factual example of the so-called “mindfulness” [62]; relatives can accept more easily the worsening of their beloved’s health conditions when they are aware that it is not related to negligence from operators [70]; when the operators are aware of the patients’ suffering, the latter may release part of their frustration [71]; sharing one’s frustration with colleagues is a convenient release-valve [72,73].

To the best of our knowledge, there are no studies in the literature that explicitly distinguish between feelings experienced during MD or afterward, which our study contributes to highlight; building on qualitative research methods, the results are not generalizable, but applicable to similar contexts [74].

## 5. Limitations

The study could not explore the experience of professional caregivers within home care settings, which is a signal for further contributions in literature. Filling this gap would be interesting, especially in light of the poor communication strategies that these operators may have available, with the following impossibility of sharing worries and frustrations. Furthermore, this study is the first to explore MD in hospital and nursing home; thus, it is not possible to extend the results to other regions or countries.

## 6. Conclusions

Communication is an important strategy to prevent or manage the occurrence of MD, across the many levels in which this phenomenon was described to occur. Further protective factors were identified by the study population, in part confirming what is known by the literature, in part adding new knowledge, or highlighting more precise cues for education, organizational arrangements, and research. Similar investigations can help provide background for the design of ethical tools and educational and organizational arrangements in support of those professionals who suffer from chronic conflicting emotions and MD.

The results may be useful to propose interprofessional reflections among practitioners, discussing real cases, including formal leaders [53], stimulate participation from real issues experienced by the participants themselves, and choose the most appropriate didactic techniques, coherently with the Ethics Round method [11]. In addition, promoting knowledge of MD in future professionals starting from degree courses will help decrease the gap between training and clinical reality that students and new employees often highlight [64].

## Figures and Tables

**Table 1:** Socio-demographic characteristics.

Characteristis	Sample
Gender	
Female	8
Male	5
Age	
18–34	5
35–49	2
50–69	6
Education	
High School	1
Bachelor	8
Degree	4
Marital Status	
Married/living together	7
Single	4
Separated/divorced	1
Missing	1
Children	
No children	7
1	2
2	2
Missing	2
Work Setting	
Hospital	6
Nursing Home	7
Type of contracts	
Full Time	10
Part Time	2
Missing	1
Type of work	
Certified Nursing Assistants	2
Professional Educator	1
Physiotherapist	1
Nurse	5
Physician	3
Psychologist	1
Years of work experience (average)	13.5
Years of work experience with elderly people (average)	11.16
